# Melanoma Cell Expression of CD200 Inhibits Tumor Formation and Lung Metastasis via Inhibition of Myeloid Cell Functions

**DOI:** 10.1371/journal.pone.0031442

**Published:** 2012-02-03

**Authors:** Fatemeh Talebian, Jin-Qing Liu, Zhenzhen Liu, Mazin Khattabi, Yukai He, Ramesh Ganju, Xue-Feng Bai

**Affiliations:** 1 Department of Pathology and Comprehensive Cancer Center, The Ohio State University, Columbus, Ohio, United States of America; 2 Integrated Biomedical Science Graduate Program, The Ohio State University, Columbus, Ohio, United States of America; 3 The Ohio State Biochemistry Program, The Ohio State University, Columbus, Ohio, United States of America; 4 Immunology/Immunotherapy Program, Georgia Health Sciences University, Augusta, Georgia, United States of America; McMaster University, Canada

## Abstract

CD200 is a cell surface glycoprotein that functions through engaging CD200 receptor on cells of the myeloid lineage and inhibits their functions. Expression of CD200 has been implicated in a variety of human cancer cells including melanoma cells and has been thought to play a protumor role. To investigate the role of cancer cell expression of CD200 in tumor formation and metastasis, we generated CD200-positive and CD200-negative B16 melanoma cells. Subcutaneous injection of CD200-positive B16 melanoma cells inhibited tumor formation and growth in C57BL/6 mice but not in Rag1^−/−^C57BL/6 mice. However, i.v. injection of CD200-positive B16 melanoma cells dramatically inhibited tumor foci formation in the lungs of both C57BL/6 and Rag1^−/−^C57BL6 mice. Flow cytometry analysis revealed higher expression of CD200R in Gr1^+^ myeloid cells in the lung than in peripheral myeloid cells. Depletion of Gr1^+^ cells or stimulation of CD200R with an agonistic antibody in vivo dramatically inhibited tumor foci formation in the lungs. In addition, treatment with tumor antigen specific CD4 or CD8 T cells or their combination yielded a survival advantage for CD200 positive tumor bearing mice over mice bearing CD200-negative tumors. Taken together, we have revealed a novel role for CD200-CD200R interaction in inhibiting tumor formation and metastasis. Targeting CD200R may represent a novel approach for cancer immunotherapy.

## Introduction

CD200 (also known as OX-2) is a member of the Ig super family of proteins. It contains two extracellular immunoglobulin domains and a small 19aa intracellular domain with no known signaling motifs [1]. CD200 is expressed in a variety of normal tissues including the central nervous system [2], epithelium structures in the thymus [3], retinas [4], hair follicles [5] and the lymphoid cells including B lymphocytes and activated T cells [6]. Recent studies have revealed that CD200 is also expressed in a variety of human cancer cells including human melanoma [7], ovarian cancer [8], myeloid leukemia cells [9] and malignant B cells [10].

CD200 is the ligand for a receptor, namely CD200R. The expression pattern of mouse and human CD200R is similar, with strong expression in macrophages, neutrophils and mast cells [11]. Triggering CD200R suppresses myeloid cell activity in vitro and engagement of CD200R by CD200 inhibits their activation [12]. Unlike most of the Ig superfamily receptors, CD200R lacks ITIM domains [13]. However, the 67 aa cytoplasmic tail contains 3 tyrosine residues and the third tyrosine residue is located within a NPXY motif, which is phosphorylated upon ligation of the CD200 receptor [14]. This leads to the recruitment and phosphorylation of Dok-2 and 1, which then bind to RasGAP and SHIP [14], [15], [16]. In macrophages and mast cells, this cascade has been shown to inhibit the phosphorylation of ERK, P38 and JNK [15].

CD200 appears to limit autoimmune inflammation in animal models of multiple sclerosis and arthritis [17] and lung injury caused by viral infection [18], as CD200 deficient mice were found to have a significantly increased disease severity due to hyper activation of macrophages. CD200R-deficient mice were also shown to be more susceptible to arthritis, presumably due to enhanced functions of macrophages but not T cell responses [19]. These findings indicate that CD200-CD200R interactions are involved in limiting the cellular functions of myeloid lineages of cells.

Expression of CD200 has been found in multiple types of cancer [9], [10], [20]. It is generally considered that expression of CD200 on cancer cells has a protumor effect based on the following evidence. First, in two correlation studies, CD200 mRNA expression in malignant cells has been shown to be associated with decreased survival of patients [9], [10]; Second, CD200-expressing melanoma and ovarian cancer cells downregulate Th1 cytokine production when co-cultured with allogenic leukocytes [7], [21] and anti-CD200 antibody treatment can enhance tumor rejection by peripheral blood mononuclear cells in a hu-SCID adoptive transfer model [22], [23]; Third, in a recent study, CD200 expression was found to be positively correlated with the metastatic capacity of squamous cell carcinoma [24]. While human correlation studies remain to be confirmed in other cancer types, studies focusing on regulating immune functions only focused on regulation of dendrtitic cells. Our recent study [25] has revealed that tumor expression of CD200 has a direct effect on tumor associated myeloid cells (TAMCs).

Myeloid cells are obligate partners for tumor cell migration, invasion and metastasis. Within the tumor microenvironment, TAMCs facilitate angiogenesis and extracellular matrix breakdown, promote tumor cell migration and invasion, and suppress antitumor immunity; at metastatic sites, TAMCs prepare the target tissue for arrival of tumor cells [26], [27]. Genetic ablation or depletion of macrophages and inhibition of macrophage functions have been shown to be effective in inhibiting tumor initiation and growth [28], [29], [30]. Since TAMCs are the major lineages of cells expressing CD200R [25], we hypothesize that tumor expression of CD200 inhibits the functions of TAMCs and thereby affects tumor formation and metastasis. To test this hypothesis, we have generated CD200-positive and CD200-negative B16.F10.OVA melanoma cells. Subcutaneous injection of CD200-positive B16 melanoma cells inhibited tumor formation and growth in C57BL/6 mice but not in Rag1^−/−^C57BL6 mice. However, i.v. injection of CD200-positive B16 melanoma cells dramatically inhibited tumor foci formation in the lungs of both C57BL/6 and Rag1^−/−^C57BL/6 mice. Depletion of Gr-1^+^ cells and stimulation of CD200R with an agonistic antibody in vivo inhibited tumor foci formation in the lungs. In addition, treatment with tumor antigen specific CD4 or CD8 T cells or their combination yielded a significant improvement in survival of CD200 positive tumor bearing mice. These data revealed a novel role for CD200-CD200R interaction in inhibiting tumor formation and metastasis.

## Materials and Methods

### Ethic Statement

This study and experimental protocols were approved by The Ohio State University Institutional Animal Care and Use Committee (IACUC) with permit number 2008A0093-R1.

### Mice

C57BL/6, Rag1^−/−^ C57BL6, OT1 (transgenic mice with TCR specific for H-2K^b^: OVA 258–265) and OT2 (TCR transgenic mice with TCR specific for I-A^b^:OVA 323–339) mice were purchased from Jackson laboratories. All mice were maintained and cared for in OSU laboratory animal facilities which are fully accredited by OSU IACUC.

### Generation of CD200 positive and negative B16.OVA cells

B16.F10 melanoma cells expressing the full length chicken ovalbumin (referred to as B16.OVA) has been described [31]. We have cloned the full-length cDNA of mouse CD200 into pcDNA3 (Invitrogen) expression vector and used it to transfect B16.OVA cells. The resulting hygromycin-resistant cells were selected for CD200 expression using flow cytometry. The empty pcDNA3 expression vector was used to transfect B16.OVA cells to generate B16.OVA.Ctrl cells. The generated CD200-positive or CD200-negative cells were maintained in RPMI 1640 medium (Gibco) supplemented with 5% FBS and 1% Penicillin/Streptomycin.

### Real time PCR

Quantitative real-time PCR was performed using an ABI 7900-HT sequence system (PE Applied Biosystems) with the QuantiTect SYBR Green PCR kit (Qiagen) in accordance with the manufacturer's instructions. PCR was done using previously determined conditions [32]. The following primers were used for amplifying specific genes: chicken OVA: 5′-ATC TCA AGC TGT CCA TGC AG -3′(forward) and 5′-TGC GAT GTG CTT GAT ACA GA -3′ (reverse). The HPRT gene was simultaneously amplified as endogenous control. The primers were 5′-AGCCTAAGATGAGCGCAAGT-3′ (forward) and 5′-TTACTAGGCAGATGGCCACA-3′ (reverse). Each sample was assayed in triplicate and the experiments were repeated twice. The relative amount of OVA mRNA was calculated by plotting the C*t* (cycle number) and the average relative expression for each group was determined using the comparative method (2^−ΔΔCt^).

### Establishment of subcutaneous and lung metastatic tumors

To establish subcutaneous tumors in C57BL/6 and Rag1^−/−^ mice, 1×10^5^ or 5×10^5^ cells/mouse were used for the subcutaneous injection. Development of tumors was monitored and tumors were measured for length (a) and width (b) every three days using a caliper. Tumor volumes were calculated as *ab^2^/2* [33]. To establish tumor lung metastasis, each mouse was injected with 1×10^5^ B16.OVA.CD200 or B16.OVA.Ctrl cells via the tail vein. Mice were monitored up to 3–4 weeks depending on symptoms and treatments received. At the end of the experiments, mice were sacrificed and lungs were collected, weighed and their tumor foci counted.

### Treatment of mice with established tumors

For T cell therapy of mice with established tumors, spleen and lymph node cells of OT1 and/or OT2 mice were incubated with a cocktail of antibodies (anti-CD4 mAb GK1.5 or anti-CD8 mAb TIB210 and anti-FcR mAb 2.4G2). After removal of unbound antibodies, the cells were incubated with anti-IgG coated magnetic beads (Dynal Biotech). Antibody coated cells were then removed using a magnet. The unbound cells consisted of >90% CD4 or CD8 T cells. Purified CD4 (5×10^6^/mouse) or CD8 (5×10^6^/mouse) or their combination (10×10^6^/mouse) were injected i.v. into mice with established tumors.

For antibody treatment of mice with lung metastatic melanoma, anti-CD200R mAb (OX110, Biolegend) or an isotype matched control IgG mAb was injected into each mouse i.v. at a dose of 100 µg/mouse every 3 days, starting on day 0 for up to five times. For depletion Gr1^+^ cells in mice, each mouse was injected 250 µg of anti-Gr1 antibody i.p. at 4 day intervals, starting on day 0.

### Antibodies and flow cytometry

For CD200 and CD200R staining, PE-labeled anti-CD200 (clone OX-90) and FITC-labeled anti-CD200R (OX-110) antibodies (Serotech) were used. FITC-, PE-, APC- or PercP- labeled antibodies to CD4, CD8α, CD11b, Gr1, Vα2, Vβ5.1/5.2 and isotype-matched control antibodies were purchased from BD Biosciences. Cells were incubated with antibodies in 0.1 M PBS (pH7.4) supplemented with 1% FCS and 0.1% sodium azide on ice for 30 minutes. They were then washed three times and fixed in 1% paraformaldehyde followed by flow cytometry analysis.

### Isolation of CD11b^+^ and Gr1^+^ cells from spleens or lungs

Mononuclear cells were prepared from spleens and lungs of C57BL/6 mice as described [34]. Briefly, minced mouse spleens and lungs were digested in collagenase at 37°C for 90 min and then filtered through a sterile nylon mesh. Myeloid cells were isolated from spleens or lungs by first staining the cell suspensions with PE-anti-CD11b mAb (BD biosciences) or PE-anti-Gr1 mAb (BD biosciences), followed by magnetic antibody cell separation using anti-PE microbeads (Miltenyi Biotec). The isolated cells were >90% pure. These cells were co-cultured with B16.OVA.Ctrl or B16.OVA.CD200 cells (1∶1 ratio), with or without LPS stimulation (100 ng/ml). Culture supernatants were collected at 24 and 48 hours for cytokine analysis.

### Cytokine ELISA

Standard ELISA procedures were used to detect IL-6, IL-10 and TNF-α in a variety of culture supernatants. All ELISA kits were purchased from eBiosciences.

### Statistical analysis

Student's t test was used to compare tumor size and number differences between two groups. For comparison of mice survival, Kaplan-Myeier survival analysis and log-rank test were used (version 10.0, SPSS, Inc., Chicago, IL). A p value less than 0.05 was considered significant.

## Results

### 1. Expression of CD200 on melanoma cells inhibits tumor formation and lung metastasis

**Figure 1:**
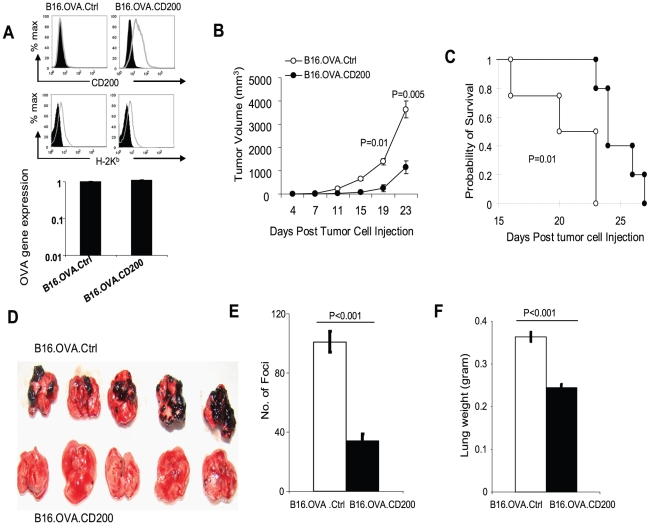
CD200 on melanoma cells inhibits tumor formation and lung metastasis. **A.** Flow cytometery analysis of B16.OVA.Ctrl and B16.OVA.CD200 cells for CD200, MHC class I H2-K^b^ expression. qRT-PCR was used to examine OVA gene expression. **B.** 1×10^5^ of B16.OVA.Ctrl or B16.OVA.CD200 cells were injected into each mouse subcutaneously. The tumor growth was observed over time. **C.** Kaplan-Meier survival analysis and log-rank test were used to analyze mice survival. Mice with a tumor burden of 1.5×1.5 cm were sacrificed and counted as dead. Data shown in **B** and **C** represent two experiments with similar results. **D.** C57BL/6 mice were given 1×10^5^ B16.OVA.Ctrl or B16.OVA.CD200 cells per mouse via their tail vein. 20 days later mice were sacrificed and tumor growth in the lungs were shown. **E.** Average number of tumor foci in the lungs from each group of mice shown in **D**. Error bars represent Mean ± SEM. Student's two-tailed t test was used for the statistical analysis. **F.** Average weight of lungs from each group of mice shown in **D**. Data shown in **D–F** represent two experiments with similar results.

Recent studies have revealed that CD200 is frequently expressed on human melanoma cells. To test the significance of melanoma expressed CD200 in tumor formation and metastasis, we generated CD200-positive and CD200-negative B16 melanoma cells by transfecting the B16.OVA cells with either the empty pCDNA3 expression vector or one carrying the murine CD200 cDNA. The resulting cells were named as B16.OVA.CD200 and B16.OVA.Ctrl, respectively. As shown in **Figure 1A**, B16.OVA.CD200 cells expressed significant levels of CD200 while the control cells were CD200 negative. Both cell types also had similar levels of MHC class I (H-2K^b^) expression (**Figure 1A**, middle panel) and similar levels of OVA gene expression (**Figure 1A**, lower panel). To examine the impact of CD200 expression on tumor formation and growth, we injected B16.OVA.CD200 or B16.OVA.Ctrl cells into C57BL/6 mice subcutaneously (s.c.). As shown in **Figure 1B** and **Figure 1C**, expression of CD200 significantly inhibited tumor formation and growth, and promoted survival of tumor bearing mice. Since B16.F10 tumors are highly metastatic to the lung, we examined tumor growth in the lung metastasis model. For this purpose, we injected C57BL/6 mice with 1×10^5^ of B16.OVA.CD200 or B16.OVA.Ctrl cells via their tail vein. On day 20, we sacrificed all mice and extracted lungs from all mice and compared their weight and tumor foci formation. As demonstrated in **Figure 1D**, the lungs from mice who received B16.OVA.Ctrl cells exhibited extensive formation of black foci, characteristic of melanoma metastasis in the lungs. In contrast, the lungs from mice injected with B16.OVA.CD200 cells had much less melanoma foci, and the differences were highly significant in terms of total foci numbers and total lung weight (**Figure 1E** **and** **Figure 1F**). These results suggest that CD200 expression on melanoma cells significantly inhibit tumor formation and lung metastasis.

### 2. Tumor expression of CD200 inhibits melanoma lung metastasis through inhibition of Gr-1^+^ myeloid cells

**Figure 2:**
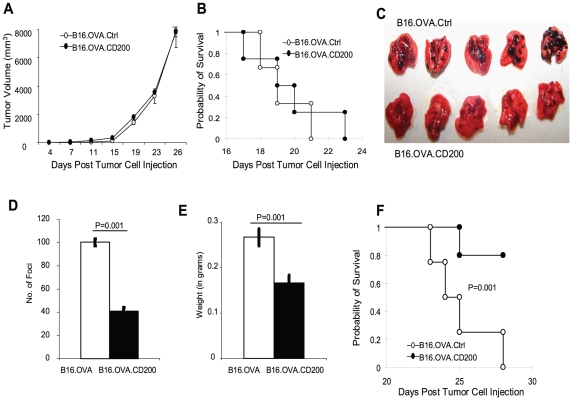
CD200 expression on tumor cells inhibits tumor lung metastasis in Rag1^−/−^ C57BL6 mice. **A.** 1×10^5^ of B16.OVA.Ctrl or B16.OVA.CD200 cells were injected into each Rag1^−/−^ C57BL6 mouse s.c. The tumor growth was observed over time. **B.** Kaplan-Meier survival analysis and log-rank test were used to analyze mice survival. Mice with a tumor burden of 1.5×1.5 cm were sacrificed and counted as dead. Data shown in **A** and **B** represent two experiments with similar results. **C.** Rag^−/−^ C57BL6 mice were given 1×10^5^ B16.OVA.Ctrl or B16.OVA.CD200 cells per mouse via their tail vein. 20 days later mice were sacrificed and tumor lung metastasis was shown. **D.** Number of tumor foci in the lungs from mice shown in **C** were quantified. Error bars represent ± SEM. Student's two-tailed t test was used for the statistical analysis. **E.** Average weight of lungs from each group of mice shown in **C**. Error bars represent ± SEM. Student's two-tailed t test was used for the statistical analysis. **F.** Kaplan-Meier survival curve of mice who received i.v. injection of B16.OVA.Ctrl or B16.OVA.CD200 cells. Data shown in **C-F** represents two experiments with similar results.

To understand if CD200-mediated inhibition of tumor formation and metastasis observed in C57BL/6 mice were due to stimulation of adaptive immunity, we did similar experiments in Rag1^−/−^ C57BL/6 mice. As demonstrated in **Figure 2A** and **Figure 2B**, expression of CD200 on melanoma cells did not significantly affect subcutaneous tumor formation and growth, and it also did not significantly affect the survival of tumor bearing mice. However, expression of CD200 on melanoma cells dramatically affected tumor foci formation in the lungs. As demonstrated in **Figure 2C** and **Figure 2D**, the lungs from mice who received B16.OVA.CD200 cells had much less melanoma foci compared with the lungs from mice that received B16.OVA.Ctrl cells (**Figure 2D**). In addition, the weight of the lungs and the survival times of mice were also significantly different between the two groups of mice (**Figure 2E** and **Figure 2F**). The results presented in **Figure 1** and **Figure 2** suggest that while adaptive immunity may play a role in the subcutaneous tumor model, the difference in tumor lung metastasis was mainly caused by innate immune components.

**Figure 3:**
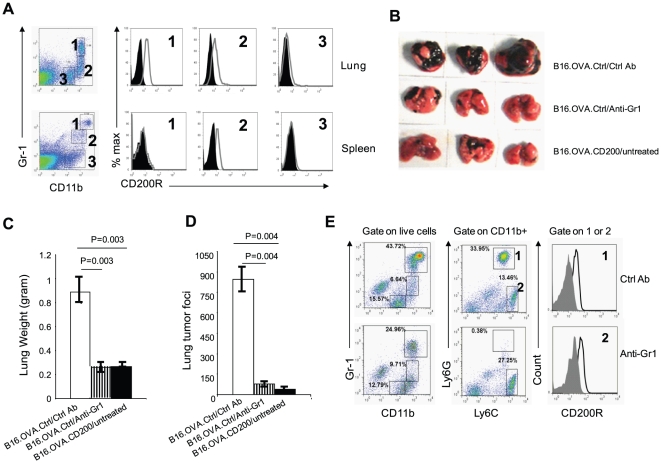
Gr1^+^ myeloid cells express CD200R and mediate lung metastasis. **A.** Flow cytometry analysis of CD200R expression in mononuclear cells from spleens and lungs. Cells were prepared from spleen and lung of normal C57BL/6 mice and were stained for CD11b, Gr1 and CD200R. The experiment was confirmed in three independent experiments with similar results. **B.** Two groups of C57BL/6 mice were injected with B16.OVA.Ctrl cells (1×10^5^ cells/mouse) via their tail vein. Mice were treated with either 250 µg/mouse of anti-Gr-1 (RB6-8C5, BioXcell) or an isotype-matched control antibody (anti-KLH, BioXcell). An untreated group of mice that received 1×10^5^/mouse of B16.OVA.CD200 cells were used for the comparison. 21 days after tumor cell injection, tumor lung metastasis was examined. **C.** Lung weight in groups of mice shown in **B**. Error bars represent ± SEM. Student's two-tailed t test was used for the statistical analysis. **D.** Average number of tumor foci in the lungs from each group of mice shown in **B**. Error bars represent ± SEM. Student's two-tailed t test was used for the statistical analysis. **E.** Flow cytometry analysis of lung mononuclear cells from anti-Gr-1 treated and control antibody treated mice. C57BL6 mice received either anti-Gr-1 (250 µg×3 doses per mouse) or isotype-matched control antibody i.p. Data shown represent three experiments with similar results.

Since tumor expression of CD200 differentially affect tumor formation in the subcutaneous model versus the lung metastatic model, we hypothesized that the lung and peripheral tumor microenvironment decided the differential susceptibility to CD200-mediated suppression of tumor growth. During tumor initiation, subcutaneous tumors mainly attract myeloid cells from blood, while in the lung, high numbers of local myeloid cells exist. We therefore compared splenic myeloid cells and those from the lungs for the expression of CD200R. As shown in **Figure 3A**, in the lung and spleens of mice, we found three populations of myeloid cells in each organ: CD11b^hi^Gr1^hi^, CD11b^hi^Gr1^lo^ and CD11b^lo^Gr1^−^ cells. In the lungs, CD11b^hi^Gr1^hi^ and CD11b^hi^Gr1^lo^ cells are the cells that express high levels of CD200R, while CD200R expression on CD11b^lo^Gr-1^−^ cells were low. In the spleens, CD11b^hi^Gr1^lo^ cells expressed lower levels of CD200R, while the other two populations of cells were essentially CD200R negative. Thus, only one minor subset of systemic myeloid cells express low levels of CD200R, while two subsets of Gr1^+^ lung myeloid cells express high levels of CD200R.

Since CD200R-positive cells also co-expressed Gr1, we tested if depletion of Gr1 positive cells could affect melanoma lung metastasis. We injected two groups of Rag1^−/−^ mice with B16.OVA.Ctrl tumor cells i.v. at a dose of 1×10^5^/mouse. One group of the mice were treated with anti-mouse Gr1 mAb i.v. every 4 days to deplete the Gr1^+^ cells. Another group of mice were treated with an isotype-matched control mAb. In parallel, a third group of mice received B16.OVA.CD200 tumor cells i.v. at a dose of 1×10^5^/mouse without mAb treatment. As demonstrated in **Figure 3B–D**, anti-Gr1 treatment dramatically reduced tumor foci in the lungs of mice that received B16.OVA.Ctrl cells. The lung weight and number of tumor foci in the lungs of anti-Gr1 treated mice were similar to the mice that only received B16.OVA.CD200. To determine if anti-Gr-1 treatment deleted CD200R-positive myeloid cells, we analyzed lung mononuclear cells isolated from anti-Gr-1 treated and control antibody treated mice. As shown in **Figure 3E**, anti-Gr-1 treatment mainly deleted a large population of Gr-1^hi^CD11b^+^ cells, which were also Ly6G^+^ and CD200R^+^. This result suggests that depletion of CD200R-positive myeloid cells has similar effects to tumor expression of CD200 in inhibiting melanoma tumor metastasis to the lungs.

**Figure 4:**
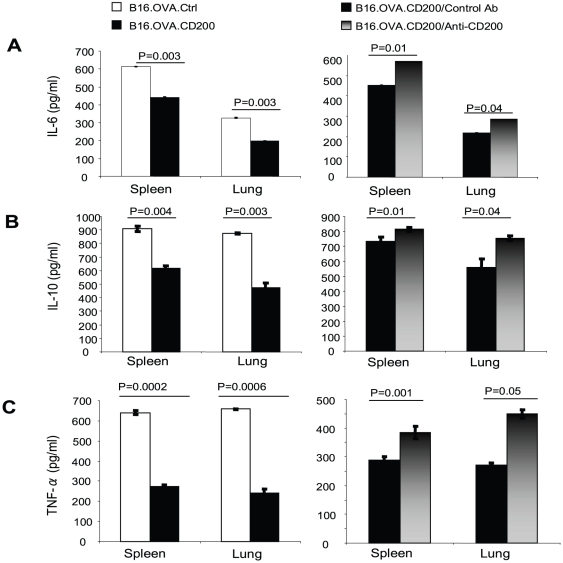
Tumor expression of CD200 inhibits the functions of Gr1^+^ myeloid cells. Gr1^+^ cells were isolated from spleens and lungs of C57BL/6 mice. The cells were then co-cultured with tumor cells at a 1∶1 ratio for 48 hours in the presence of 100 ng/ml of LPS (left panel) and 10 µg/ml of anti-CD200 mAb or an IgG2a isotype control mAb (right panel). The supernatants were collected from the co-cultures and were examined for the presence of IL-6, IL-10 and TNF-α. Experiments were repeated at least 3 times with similar results. Data shown are mean ± SEM of 5 mice. Student's two-tailed t test was used for the statistical analysis.

To determine if tumor cell expression of CD200 directly inhibits the functions of Gr1^+^ myeloid cells, we purified Gr1^+^ cells from spleens or lungs using MACS beads. We co-cultured purified splenic and lung Gr1^+^ cells with either B16.OVA.Ctrl or B16.OVA.CD200 cells in the presence of LPS. The concentrations of IL-6, IL-10 and TNF-α in the culture supernatants were measured using ELISA. As shown in **Figure 4**, cytokine levels were consistently lower in cultures containing CD200-positive tumor cells, while CD200 blockade using an anti-CD200 antibody abrogated the suppression of cytokine production (**Figure 4**, right panel). These data suggest that tumor expression of CD200 can directly inhibit the functions of Gr1^+^ myeloid cells via interaction with CD200R.

### 3. Triggering CD200R inhibits tumor foci formation in the lungs

**Figure 5:**
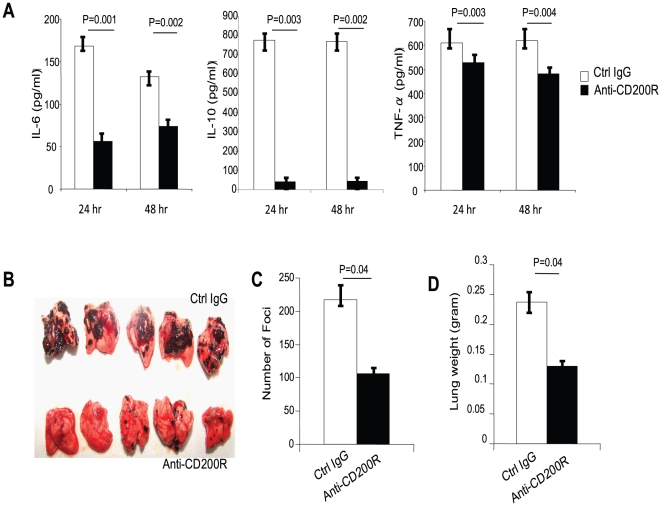
Triggering CD200R using a mAb inhibits melanoma lung metastasis. **A.** CD11b^+^ cells were isolated from the spleen of C57BL/6 mice and were stimulated with LPS (100 ng/ml) in the presence or absence of a monoclonal anti-mouse CD200R antibody (OX110). Supernatants from the cultures were collected at 24 and 48 hours and concentrations of IL-6, IL-10 and TNF-α were examined using ELISA. Data shown represents three independent experiments with similar results. Data shown are mean ± SEM from groups of three mice. Student's two-tailed t test was used for the statistical analysis. **B.** 1×10^5^ B16.OVA.Ctrl cells were injected into each C57BL/6 mice via their tail vein. Mice were treated with either 100 µg/mouse of anti-CD200R (n = 5) or an isotype-matched control antibody (n = 5) every 3 days starting from day 0. 18 days later mice were sacrificed and melanoma lung metastasis was examined in antibody-treated mice or controls. Data shown represents two experiments with similar results. **C.** Average number of tumor foci in the lungs from each group of mice. Error bars represent ± SEM. Student's two-tailed t test was used for the statistical analysis. **D.** Average weights of lungs from each group of mice. Error bars represent ± SEM. Student's two-tailed t test was used for the statistical analysis.

Our results suggest that CD200-CD200R interaction plays important roles in both innate and adaptive components in tumor immunity, therefore targeting CD200-CD200R interaction should provide a potential option for treatment of cancer. CD200 is only expressed in some lineage of tumors, while CD200R is expressed in tumor associated myeloid cells essentially in all solid tumors; thus, targeting CD200R should have broader implication in the treatment of cancer. To test this hypothesis, we tested the efficacy of an agonistic anti-CD200R mAb (OX110) [11] in the treatment of lung metastasis of CD200-negative melanoma. To test the efficacy of OX110 on myeloid cells, we isolated CD11b^+^ cells from Rag1^−/−^ C57BL/6 mice. The cells were stimulated with LPS in the presence of OX110 (Biolegend) or an isotype-matched control mAb (Biolegend). As shown in **Figure 5A**, anti-CD200R mAb significantly diminished production of cytokines by CD11b^+^ myeloid cells, suggesting that mAb OX110 can inhibits the functions of myelid cells. To test its in vivo effects on tumor foci formation and lung metastasis, C57BL/6 mice were injected with 1×10^5^ B16.OVA.Ctrl cells i.v. Starting from day 0, we treated mice with 100 µg/mouse of OX110 mAb or 100 µg/mouse of an isotype-matched control IgG i.v. every 3 days. As shown in **Figure 5B**, anti-CD200R treatment dramatically reduced melanoma tumor formation in the lungs compared to treatment with the control antibody. Numbers of melanoma foci (**Figure 5C**) and lung weights (**Figure 5D**) were also significantly different between the two groups. Thus, triggering CD200R inhibits tumor foci formation in the lungs and targeting CD200R by a triggering mAb is feasible for the treatment of CD200-negative tumors.

### 4. Tumor expression of CD200 improves the efficacy of T cell adoptive transfer therapy

**Figure 6:**
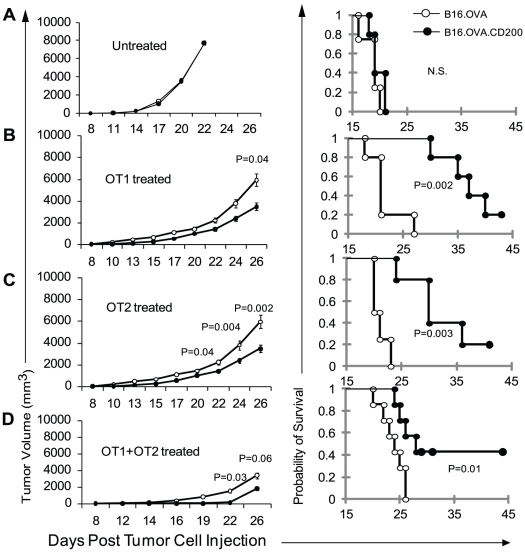
CD200-positive tumors are more susceptible to adoptive T cell therapy. **A.** 5×10^5^ of tumor cells were injected into each Rag1^−/−^ C57BL6 mouse s.c. Tumor growth (left) and survival of mice (right) were monitored over time. N = 5 mice per group and data shown represents two experiments with similar results. **B.** 5×10^5^ of tumor cells were injected into each Rag1^−/−^ C57BL/6 mouse s.c. Mice were then given 5×10^6^/mouse of purified CD8^+^ T cells from OT1 mice i.v. 5 days after tumor cell injection. Five mice per group were used and data shown represents two experiments with similar results. **C.** 5×10^5^ of tumor cells were injected into each Rag1^−/−^ C57BL/6 mouse s.c. Mice were then given 5×10^6^/mouse of purified CD4^+^ T cells from OT2 mice i.v. 5 days after tumor cell injection. Five mice per group were used and data shown represents two experiments with similar results. **D.** 5×10^5^ of tumor cells were injected into each Rag1^−/−^ C57BL/6 mouse s.c. Mice were then given 5×10^6^/mouse of purified OT1 and 5×10^6^/mouse of purified OT2 T cells i.v. 5 days after tumor cell injection. Five mice per group were used.

The differential effects of tumor expression of CD200 on subcutaneous tumor growth in C57BL/6 mice versus Rag1^−/−^ mice (**Figures 1** **&** **2**) suggest that CD200 expression on tumor cells affects adaptive immunity. We previously demonstrated that tumor expression of CD200 inhibits the functions of tumor associated myeloid cells and permits better tumor eradication by CTL [25]. To test if expression of CD200 on melanoma cells could improve the susceptibility to T cell therapy, we injected 5×10^5^ of B16.OVA.Ctrl or B16.OVA.CD200 tumor cells into each Rag^−/−^C57BL/6 mouse subcutaneously. The mice were either left untreated, or treated with 5×10^6^ CD8^+^ T cells purified from OT1 mice or CD4^+^ T cells from OT2 mice or their combination. As shown in **Figure 6**, no significant difference was observed between untreated CD200-positive and CD200-negative tumors (**Figure 6A**). Adoptive transfer of OVA-specific CD8 (**Figure 6B**), CD4 (**Figure 6C**) or their combination (**Figure 6D**) significantly improved the efficacy of T cell therapy on CD200-positive, but not on CD200-negative tumors. The tumor volumes (**left panel**) of T cell-treated CD200-positive tumors were significantly smaller and the survival times of mice with CD200-positive tumors were significantly longer compared to mice with CD200-negtive tumors receiving the same T cell treatment (**right panel**). Thus, expression of CD200 on melanoma cells improves the efficacy of T cell therapy.

## Discussion

Expression of CD200 has been implicated in a variety of human cancer cells including melanoma cells [7] and has been reported to play protumor effects via inhibiting tumor immunity [7], [22], [24]. However, using CD200-positive and CD200-negative melanoma tumor models, we have revealed a novel role for CD200-CD200R interaction in inhibiting tumor formation and metastasis, i.e. tumor expression of CD200 inhibits tumor formation and metastasis via inhibiting the functions of CD200R^+^ myeloid cells. As we showed in other tumor models [25], we further confirm here that CD200-positive melanoma tumors are more susceptible to antigen specific T cell therapy compared to CD200-negative melanoma. Thus, our data challenge the current paradigm that tumor expression of CD200 promotes tumor progression and metastasis.

Myeloid cells are pivotal in tumor initiation, tumor mass formation, tumor progression and metastasis [27]. In the tumor initiation and formation stage, myeloid cells produce an array of factors that promote tumor establishment. During the tumor progression and metastasis stages, myeloid cells provide support for developing tissues through their matrix remodeling capacities, synthesis of growth and angiogenesis factors and capacity in suppressing antitumor immunity. Genetic ablation, depletion of myeloid cells or inhibition of myeloid cell functions have been shown to be effective in inhibiting tumor establishment and tumor progression [28], [29], [30], [35]. It has been found that increased number of myeloid cells is strongly associated with shortened survival in patients with classic Hodgkin's lymphoma [36]. We have recently demonstrated that tumor associated myeloid cells express high levels of CD200R, and they are susceptible to CD200-mediated inhibition [25]. In this study, we have compiled evidence that tumor expressed CD200 can directly interact with myeloid cells to inhibit tumor formation and metastasis in a CD200R-dependent manner. In in vitro cultures, CD200-positive tumor cells but not CD200-negative tumor cells strongly suppress cytokine production by myeloid cells. Compared to peripheral myeloid cells, Gr1^+^ lung myeloid cells express much higher levels of CD200R. This correlated with profound inhibition of tumor formation and metastasis of CD200^+^ B16 melanoma in the lung while the effect was diminished in the periphery (**Figures 1** **and** **2**). In contrast to our observation, CD200 induction on tumor cells was recently shown to correlate with more tumor metastasis [24]. However, in that study it was unclear if expression of CD200 on metastatic tumors was responsible for tumor metastasis. In our current study, we have clearly shown that depletion of a large population of CD200R^+^ myeloid cells (Ly6G^+^) using anti-Gr1 mAb achieved a similar effect to CD200 expression on melanoma cells (**Figure 3**). We also demonstrated that injection of a triggering CD200R mAb dramatically inhibited tumor formation and the metastatic ability of melanoma cells to the lung. Thus, our data establish that tumor expression of CD200 inhibits tumor formation and metastasis via inhibiting CD200R^+^ myeloid cells.

Our previous study [25] has revealed that CD200-positive plasmacytoma J558 and mastocytoma P815 tumors are more susceptible to CTL adoptive transfer therapy presumably due to a more permissive tumor microenvironment. In this study we found that adoptive transfer of antigen-specific T cells (both CD4 and CD8) also significantly promoted survival of mice with CD200-positive melanoma tumors over CD200-negative tumors. Moreover, significant growth delay of CD200-positive B16 tumors in immune competent mice but not in immune-deficient Rag1^−/−^ mice suggest that adaptive immunity is also stimulated by CD200-positive tumors. These observations are controversial to the current concept that tumor expression of CD200 inhibits DC, thereby inhibiting activation of tumor antigen specific T cells [7]. In mice with established tumors, the major populations of CD200R^+^ cells are MDSC and TAMs. Since activation of T cells does not require direct interaction of whole tumor cell with DC, the concept must be tested in vivo in a definitive model. Our data support a model that tumor expression of CD200 inhibits CD200R^+^ myeloid cells in the tumor microenvironment, which renders the tumor microenvironment more permissive to T cells and confers better tumor destruction.

Given the important roles of CD200-CD200R interaction in regulating tumor associated myeloid cells and in inhibiting tumor formation and metastasis, targeting CD200-CD200R interaction should provide an option for the immunotherapy of human cancer. Because of the restricted expression of CD200R to myeloid cells and the importance of these cells in essentially all tumor types, targeting CD200R should be an ideal option. Our successful treatment of CD200-negative tumors using a triggering anti-CD200R mAb proves that this approach is feasible.
